# Methylome-driven regulation of miRNA expression and its relationship to cardiac dysfunction in idiopathic dilated cardiomyopathy

**DOI:** 10.1186/s13148-025-01989-8

**Published:** 2025-10-22

**Authors:** Alex Gallego-Martínez, Marta Delgado-Arija, Irene González-Torrent, Lorena Pérez-Carrillo, Carlota Benedicto-Marrero, Juan Bodí-Miret, Manuel Portolés, Estefanía Tarazón, Esther Roselló-Lletí

**Affiliations:** 1 Clinical and Translational Research in Cardiology Unit, Health Research Institute Hospital La Fe (IIS La Fe), Avd. Fernando Abril Martorell 106, 46026 Valencia, Spain; 2 Center for Biomedical Research Network on Cardiovascular Diseases (CIBERCV), Avd. Monforte de Lemos 3-5, 28029 Madrid, Spain

**Keywords:** Idiopathic dilated cardiomyopathy, DNA methylation, MicroRNAs, Epigenomics

## Abstract

**Background:**

Idiopathic dilated cardiomyopathy (iDCM) is a multifactorial disease with a complex pathogenesis involving diverse molecular mechanisms. Among these, epigenetic mechanisms, including both DNA methylation and microRNAs (miRNAs)-mediated regulation, play an important role in determining the disease phenotype. However, the interplay between the DNA methylome and the miRNA transcriptome in iDCM remains largely unexplored.

**Methods:**

We conducted a cross-cohort multiomic integrative analysis of left ventricular (LV) tissue samples from iDCM patients and control (CNT) donors. DNA methylation profiling was performed using the Infinium MethylationEPIC BeadChip, whereas ncRNA-seq was used to assess transcriptomic changes.

**Results:**

We identified a subset of three miRNAs exhibiting both differential methylation in their promoter regions and differential expression in their primary and mature forms. Notably, the miRNA hsa-miR-433-3p (*r* = 0.671, *p* < 0.01), which is involved in fibrotic pathways, appear to be significantly correlated with the left ventricular ejection fraction (LVEF), an established echocardiographic marker of cardiac function.

**Conclusions:**

This study enhances our understanding of the epigenetic mechanisms shaping the miRNA transcriptomic landscape in iDCM, suggesting potential roles for these miRNAs in cardiac dysfunction and myocardial fibrosis.

**Supplementary Information:**

The online version contains supplementary material available at 10.1186/s13148-025-01989-8.

## Background

Dilated cardiomyopathy (DCM) is a progressive disease of the myocardium that involves structural and functional alterations, eventually leading to heart failure (HF) and other cardiac complications [1]. It is characterized by an enlargement of the ventricular chamber that is accompanied by a systolic dysfunction and a reduction in the left ventricular ejection fraction (LVEF) without the presence of coronary artery disease, hypertension, valvular disease, or congenital heart disease sufficient to cause the observed myocardial abnormality [2].

Among the different clinical subtypes classified by the European Society of Cardiology [3], idiopathic DCM (iDCM) has attracted interest because of its prevalence, and is a major reason for cardiac transplantation in children and adults [4, 5]. In contrast to some familial forms of DCM, in which there is a known genetic cause for the observed heart alterations, the aetiology behind iDCM is heterogeneous, and several mechanisms have been proposed to be key in its development [6–13].

In this context, epigenetic modifications have gained attention for their role in regulating gene expression and shaping the cellular phenotype, thereby contributing to both the maintenance of homeostasis and disease onset and progression [14, 15]. Functionally, epigenetics involves both structural and regulatory mechanisms, including DNA methylation, histone modifications, and the activity of noncoding RNAs (ncRNAs) [16]. DNA methylation involves the addition of methyl groups to cytosine-guanine (CpG) sites in the genome by DNA methyltransferases (DNMTs), modulating gene expression and influencing disease development [17]. Previous studies have shown that altered DNA methylation patterns in specific genomic regions of iDCM heart tissue are directly associated with the development of the disease by modulating the expression of key genes involved in its pathogenesis [18, 19].

These structural epigenetic alterations can occur throughout the genome and influence the expression of both coding and noncoding regions [20]. Transcription within the latter gives rise to ncRNAs, a diverse group of transcripts that exert their function without being translated into proteins [21], conferring an additional regulatory epigenetic layer. Among these, microRNAs (miRNAs) are small molecules, 21–23 nucleotides in length, that play a key role in posttranscriptional gene regulation [22]. They are derived from primary transcripts known as pri-miRNAs, which are posttranscriptionally processed into precursor miRNAs (pre-miRNAs) before maturing into functional miRNAs [23]. Notably, miRNAs have been identified as critical regulators of the onset and progression of cardiovascular diseases, including various forms of cardiomyopathy [24–27].

The epigenetic regulation of miRNA expression through DNA methylation mechanisms has recently been proposed as an additional layer for the complex determination of the cellular transcriptome [28], and its impact on the miRNA expression profile has been explored in pathologies such as cancer [29–33] and pulmonary fibrosis [34]. However, there is no information about the impact of these mechanisms in the context of iDCM. In this study, we performed an integrated transcriptomic and epigenomic analysis of left ventricular (LV) tissue samples from iDCM patients to investigate whether a relationship exists between DNA methylation and miRNA expression in the context of this disease. Furthermore, we conducted correlation analyses with echocardiographic parameters to assess potential links between these molecular alterations and cardiac function.

## Methods

### Cardiac tissue samples and patients

Myocardial tissue samples were collected from the LV apex of patients diagnosed with iDCM undergoing heart transplantation, as well as from control donors (CNT). The tissue samples were preserved in 0.9% NaCl at 4 °C for an average of 4.4 ± 3 h following coronary circulation loss and then stored at − 80 °C until further analysis. Ischaemia time could be a critical factor for both epigenomics and transcriptomics. However, DNA methylation is relatively stable against post-mortem degradation or processing delays [35], allowing for reliable DNA methylation analysis. RNA is much more labile than DNA, and ischaemia time can affect its quality. Nevertheless, the RNA integrity number (RIN) of these samples is good (≥ 9.0), with no differences found between the control and patient groups (*p* = 0.562).

The epigenomic profile of the LV samples was assessed using two complementary approaches: an Infinitum MethylationEPIC BeadChip epigenetic analysis involving 15 LV samples (iDCM: *n* = 6; CNT: *n* = 9), and a ncRNA sequencing (ncRNA-seq) analysis of 28 samples (iDCM: *n* = 20; CNT: *n* = 8).

Patients were diagnosed on the basis of their clinical history and findings from haemodynamic, electrocardiographic, Doppler echocardiography, and coronary angiography studies. All patients were classified according to the New York Heart Association (NYHA) functional criteria and received medical treatment following the European Society of Cardiology guidelines [36]. The iDCM diagnosis was established on the basis of a LVEF < 40% and a left ventricular end-diastolic diameter (LVEDD) > 55 mm, as determined by echocardiography, in the absence of a history of typical angina pectoris or myocardial infarction. CNT samples were obtained from nondiseased hearts deemed unsuitable for transplantation due to blood type incompatibility or surgical constraints. The causes of death of the donors were either motor vehicle accidents or cerebrovascular events.

### DNA extraction and quality assessment

Twenty milligrammes of frozen LV tissue (iDCM: *n* = 6; CNT: *n* = 9) were homogenized in a buffer containing 10 mM EDTA, 200 mM NaCl, and 10 mM Tris–HCl (pH 7.4), along with 10% SDS, and proteinase K (20 mg/mL). The mixture was incubated overnight at 37 °C with continuous stirring. The homogenates were then heated at 75 °C for 15 min and treated with RNase A (10 mg/mL) for 1.5 h at 37 °C.

DNA was extracted using a modified phenol–chloroform protocol, as previously described [37]. The extraction products were quantified using the Quant-iT PicoGreen dsDNA Assay (Life Technologies, Carlsbad, CA, USA), and DNA purity was assessed using a NanoDrop 1000 spectrophotometer (Thermo Scientific, Waltham, MA, USA) by measuring the 260/280 and 260/230 ratios. DNA integrity was evaluated by electrophoresis on a 1.3% agarose gel.

### Infinitum MethylationEPIC BeadChip analysis

Epigenomic analysis was conducted using the Infinium MethylationEPIC BeadChip platform (Illumina, San Diego, CA, USA) to assess over 850,000 CpG sites [38]. This platform utilizes the Infinium HD chemistry assay, which is shared with the HumanMethylation450 BeadChip. Consequently, the MethylationEPIC protocol follows the same steps as the HumanMethylation450 protocol, as previously described [39].

The purified DNA samples were randomly distributed into 96-well plates. Bisulfite conversion of 600 ng of genomic DNA was performed using the EZ-96 DNA Methylation Kit (Zymo Research Corp., Irvine, CA, USA) according to the manufacturer’s instructions. Approximately 200 ng of bisulfite-converted DNA was then hybridized onto the Infinium MethylationEPIC BeadChip. Briefly, the workflow included whole-genome amplification, enzymatic endpoint fragmentation, precipitation, and resuspension. The resuspended samples were hybridized onto the BeadChip for 16 h at 48 °C, followed by washing. Single-nucleotide extension with labelled dideoxynucleotides was performed, followed by multiple rounds of staining using a combination of labelled antibodies against biotin and 2,4-dinitrophenol.

To ensure data consistency, colour balance adjustment and quantile normalization were applied to normalize the samples between the two-colour channels. DNA methylation levels are expressed as beta values (*β*) ranging from 0 to 1.

### RNA extraction and integrity assessment

Myocardial samples were homogenized using a TissueLyser LT (Qiagen, Hilden, Germany). RNA extraction for ncRNA-seq analysis was performed using a Quick-RNA™ Miniprep Plus Kit (Zymo Research, CA, USA) in 28 samples (iDCM: *n* = 20; CNT: *n* = 8). RNA quantification was conducted using NanoDrop 1000 spectrophotometer and a Qubit 3.0 fluorometer (Thermo Fisher Scientific, Horsham, UK). RNA purity and integrity were assessed using a 0.8% agarose gel electrophoresis and Agilent 2100 Bioanalyzer with the RNA 6000 Nano LabChip Kit (Agilent Technologies, CA, USA). All the samples presented a 260/280 absorbance ratio > 2.0 and an RNA integrity number ≥ 9.0.

### ncRNA-seq analysis

For the ncRNA-seq analysis, cDNA libraries were prepared according to Illumina´s recommendations. Briefly, 3′ and 5′ adaptors were sequentially ligated to the RNA before reverse transcription and cDNA synthesis. The resulting cDNA was amplified using PCR to generate an indexed double-stranded cDNA library, followed by size selection with a 6% polyacrylamide gel. Library quality and concentration were assessed using a 4200 TapeStation (Agilent Technologies; Madrid, Spain) with the D1000 high-sensitivity assay. The cDNA libraries were then pooled and sequenced using paired-end sequencing (100 × 2) on an Illumina HiSeq 2500 sequencer (Illumina, CA, USA).

The quality control of the raw data was performed using the FastQC tool. For the adapter and quality filler of the raw data, trim_galore was applied [http://www.bioinformatics.babraham.ac.uk/projects/trim_galore/] (accessed on 15 May 2020). Then, the insufficient quality reads (phred score < 20) were subsequently eliminated using Picard Tools software [40]. RNA predictions were estimated using HT Seq software (version 0.6.0) [41].

### Data analysis

The results for each continuous variable were tested for normality using the Shapiro–Wilk method, and they are presented as the means ± standard deviations (variables with a normal distribution) or medians and interquartile ranges (nonnormal distributions). Continuous variables not normally distributed were compared using the Mann–Whitney test, and variables with a normal distribution were compared using Student’s *t*-test. Finally, Pearson’s or Spearman’s correlation coefficient was calculated to determine the statistical correlation between variables. Categorical variables are presented as percentages and Fisher’s exact test was used to assess the distribution similarity between groups.

For the methylation analysis, differentially methylated CpG positions (DMPs) were identified and mapped to their genomic locations using coordinate data from the Illumina Database (Infinium MethylationEPIC v1.0 B5 Manifest File, March 13, 2020). For the analysis, a linear model was used, incorporating sex as a covariate. CpG sites with a high sex-dependency effect were excluded, and the beta values of the remaining sites were adjusted to discriminate between patients and controls, regardless of sex. To increase statistical power, the t-statistics were moderated using an empirical Bayesian approach. The false discovery rate (FDR) correction using the Benjamini–Hochberg (BH) method was applied, considering CpG sites with an FDR < 0.05 as significant. The *β* difference (∆*β*) value for each DMP was calculated by subtracting the median *β* value of the CNT group from that of the iDCM group, and those with |∆*β*|≥ 0.10 were selected for further analysis. To explore the relationship between epigenomic alterations and miRNA expression, we assessed the methylation status of promoter regions associated with significantly differentially expressed miRNAs. The transcription start site (TSS) coordinates for the selected miRNAs were obtained from the miRStart 2.0 database [42], and the highest-scoring predictions were selected. Promoter regions were defined as the ± 20,000 base pair (bp) region flanking each TSS, according to the methodological approach of similar published studies [43, 44]. DMPs located within these promoter regions were identified for further analysis to investigate potential epigenetic regulatory effects on miRNA expression.

pri-miRNA and mature miRNA expression analysis was conducted by comparing the expression levels between the iDCM and CNT groups. The raw sequencing reads were processed, and normalized expression values were obtained using the appropriate bioinformatics pipeline. Differential expression analysis was performed to identify miRNAs and pri-miRNAs whose expression significantly changed, and the fold change (FC) was calculated as the ratio of the mean expression levels between groups. The statistical significance of differential expression was determined using an appropriate statistical test, generating *p* values to assess the likelihood of observed differences occurring by chance. We used the FDR method to adjust the original *p* value using the number of tests. To facilitate interpretation, FC values were log-transformed (log2FC), with positive values indicating upregulation and negative values indicating downregulation in iDCM samples compared with controls. Additionally, *p* adjusted values were transformed to − log10 (*p* adj) to better visualize statistical significance, where higher values indicate greater significance. Pri-miRNAs and mature miRNAs were classified as differentially expressed if they met both the log2FC (≤ − 0.6 or ≥ 0.6) and FDR < 0.05 cutoff. miRNA target identification was performed using data obtained from the miRDB [45] database (accessed in March, 2025), applying a target score threshold of ≥ 95, to ensure high-confidence interactions for downstream analysis and TargetScanHuman version 8.0 [46]. A functional enrichment analysis of differentially methylated genes based on hypergeometric testing using the ToppGene suite was performed [47]. All data analyses were conducted using R version 4.3.3 (R Core Team, Vienna, Austria), and figures were constructed using GraphPad Prism 8.

## Results

### Clinical traits of patients

The methylation analysis included 15 LV tissue samples (6 iDCM, 83% male and a mean age of 57 ± 6.4 years, and 9 CNT, 65% male with a mean age of 55 ± 17 years), whereas the ncRNA-seq analysis included 28 samples (20 iDCM, 85% male and a mean age of 50 ± 11 years, and 8 CNT, 29% male and a mean age of 51 ± 15). The iDCM patients’ clinical characteristics, including demographics, clinical history, and echocardiographic variables, are summarized in Table 1.

**Table 1:** Clinical characteristics of the included patients

	Methylation (*n* = 6)	ncRNA-seq (*n* = 20)	*p* value
*Clinical history variables*			
Gender male (%)	83	85	1.0
Age (years)	57 ± 6.4	50 ± 11	0.20
BMI (kg/m^2^)	25 ± 3.3	26 ± 4.1	0.81
Systolic blood pressure (mmHg)	107 ± 12	114 ± 14	0.37
Diastolic blood pressure (mmHg)	73 ± 8.2	73 ± 10	0.99
Haemoglobin (g/dL)	13 ± 1.0	13 ± 2.5	0.94
Haematocrit (%)	41 ± 3.2	39 ± 6.3	0.38
Total cholesterol (mg/dL)	158 ± 53	145 ± 39	0.72
HDL (mg/dL)	25 ± 18	32 ± 16	0.60
LDL (mg/dL)	70 ± 59	76 ± 28	0.82
VLDL (mg/dL)	26 ± 11	18 ± 7.8	0.29
Prior hypertension (%)	16	20	1.0
Prior hypercholesterolemia (%)	0.0	10	1.0
Prior smoking (%)	50	55	1.0
Diabetes mellitus (%)	0.0	10	1.0
Duration of the disease (months)	56 ± 46	58 ± 43	0.94
*Echocardiographic study*			
LVEF (%)	19 ± 8.2	18 ± 7.7	0.90
LVESD (mm)	66 ± 8.6	69 ± 11	0.61
LVEDD (mm)	74 ± 7.4	77 ± 9.1	0.51
LVMI (g/cm^2^)	431 ± 38	440 ± 83	0.89

BMI, body mass index; HDL, high-density lipoprotein; LDL, low-density lipoprotein; VLDL, very-low-density lipoprotein; LVEF, left ventricular ejection fraction; LVESD, left ventricular end-systolic diameter; LVEDD, left ventricular end-diastolic diameter; LVMI, left ventricular mass index; duration of disease, from heart failure diagnosis until heart transplant. Qualitative data are presented as percentages, and quantitative data are presented as the means ± standard deviations

### Epigenetic analysis

**Fig. 1:**
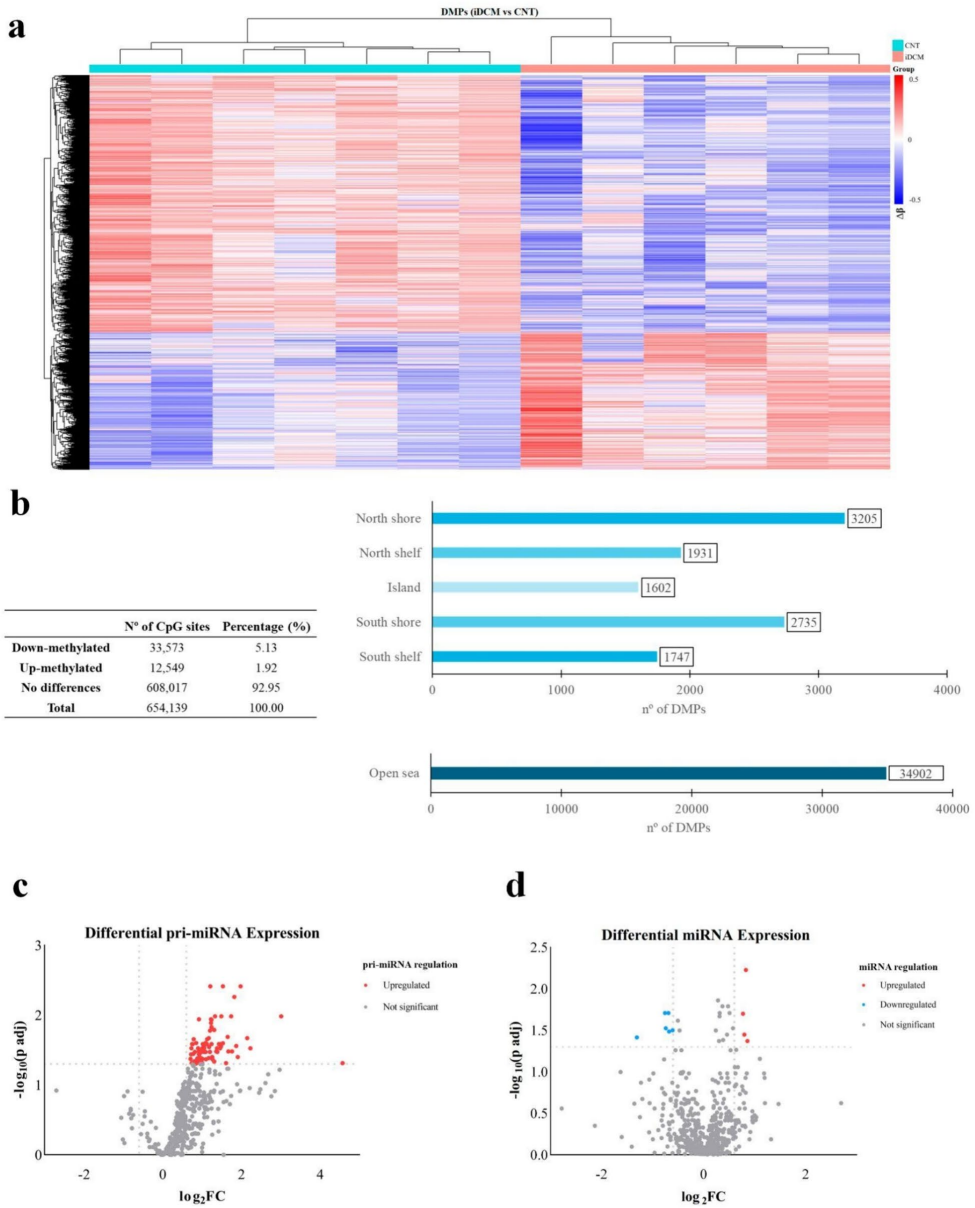
Epigenetic analysis of LV tissue samples. **a** Heatmap of DMPs comparing the iDCM and CNT LV samples, highlighting a greater prevalence of hypomethylated CpG sites (blue) in the iDCM samples relative to CNT. **b** Analysis of the state and distribution of the DMPs in the iDCM sample group. Volcano plot of the differentially expressed pri-miRNAs, **c**, and miRNAs, **d**, as assessed by RNA sequencing analysis in the LV tissue of patients with iDCM compared with that in the CNT samples. The y-axis shows the − log10 of the adjusted *p* values, and the *x*-axis indicates the FC measured as the log2−transformed ratio of the expression between both experimental groups. DMPs, differentially methylated positions; CNT, control group; iDCM, idiopathic dilated cardiomyopathy

The methylation status of the CpG sites in the iDCM samples was assessed by comparing them to the CpG sites in the CNT samples. Genome-wide methylation microarray analysis revealed 46,122 DMPs in the iDCM group (see Additional File 1), with hypomethylation (33,573 CpG sites) being more prevalent than hypermethylation (12,549 CpG sites) (*p* < 0.05) (Fig. 1a, b). Figure 1b shows the distribution of the differentially methylated CpG sites by region in iDCM patients.

ncRNA transcriptomic analysis was used to assess the expression of 514 pri-miRNAs. Among these, 88 pri-miRNAs exhibited significant differential expression in the iDCM LV tissue samples, all of which were found to be upregulated (Fig. 1c; see Additional File 2). The analysis of 655 mature miRNAs revealed a differential expression of 11 miRNAs, 7 of which were downregulated and 4 of which were upregulated (Fig. 1d, see Additional File 3). Target genes for these miRNAs were identified using two established resources, miRDB [45] and TargetScanHuman [46]. Only genes predicted by both databases were retained as high-confidence targets (see Additional File 4). In addition, a cross-cohort multiomic integrative analysis of LV tissue samples from iDCM patients and CNT donors was realized to accomplish a functional enrichment of the differentially methylated genes targets of differentially expressed miRNAs. Additional File 5 shows the main biological process, molecular function and cellular component in which the differentially methylated genes are included.

Further analysis identified four differentially expressed pri-miRNAs whose expression patterns were consistent with those of four of the differentially expressed mature miRNAs, with the expression patterns consistent among both mature and immature forms (Table 2).

**Table 2:** Differentially expressed mature miRNAs and their corresponding pri-miRNAs in iDCM LV tissue samples

pri-miRNA	FC	log_2_FC	*P* adj	− log_10_(*p* adj)	miRNA	FC	log_2_FC	*P* adj	− log_10_(*p* adj)
MIR130B	3.43	1.78	0.01	1.83	hsa-miR-130b-5p	1.77	0.83	0.01	2.23
MIR195	2.51	1.33	0.03	1.47	hsa-miR-195-3p	1.71	0.77	0.02	1.70
MIR433	3.49	1.80	0.01	1.93	hsa-miR-433-3p	1.74	0.80	0.04	1.45
MIR760	3.50	1.81	0.03	1.53	hsa-miR-760	1.81	0.86	0.04	1.37

FC, fold change in the idiopathic dilated cardiomyopathy group compared with the control group

### Methylation and miRNA transcriptomic data integration

To investigate whether a relationship between the epigenomic state and miRNA transcriptomic expression exists, the methylation state of the defined promoter regions of the significantly differentially expressed pri-miRNAs was analysed using TSS location information extracted from the miRStart 2.0 database [42]. This analysis revealed that 3 out of the 4 differentially expressed pri-miRNAs, corresponding to 3 mature miRNAs, presented at least 1 DMP in the selected promoter area (Table 3). Additionally, the calculated average beta difference (*A*∆*β*) showed a reduction in the methylation status of the promoter regions, for *hsa-miR-195,* whereas the promoter regions, for *hsa-miR-433,* showed an increase in methylation. Notably, *hsa-miR-760* displayed both increased and decreased methylation across different promoter-associated regions.

**Table 3:** DMPs in the promoter regions of differentially expressed miRNAs

miRNA	DMPs in promoter region of miRNA	*A*∆*β*
MIR195	cg01216311	− 0.11
MIR433	cg09212014	0.10
MIR760	cg23933261	− 0.10
	cg16736841	0.11
	cg01450441	0.12

*A*∆*β*, average beta difference (control vs. idiopathic dilated cardiomyopathy); DMPs, differentially methylated positions

### The relationship between cardiac function and differentially expressed and methylated miRNAs

**Fig. 2:**
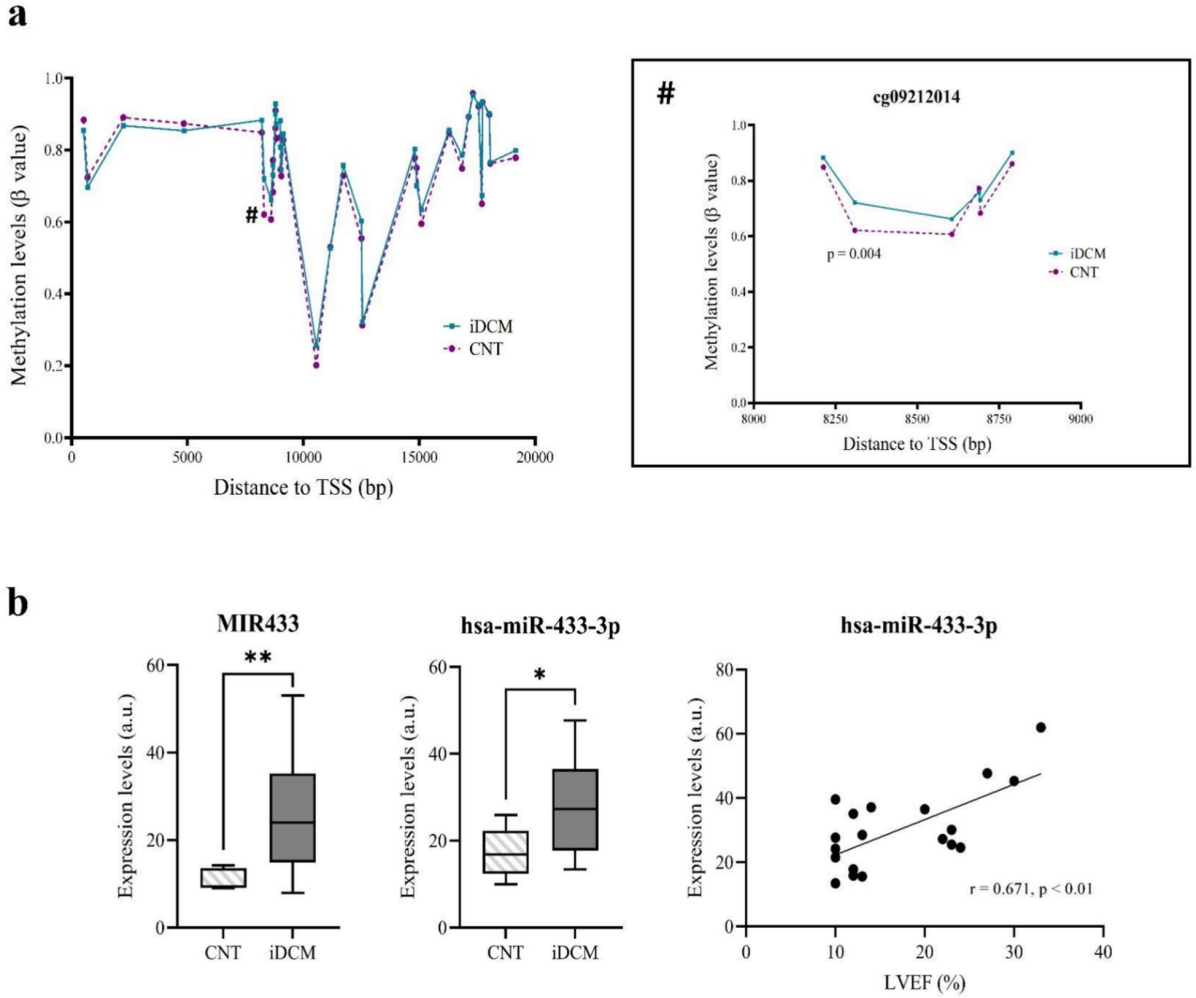
Associations between cardiac function and differentially expressed/methylated miRNA. **a** Methylation pattern of the MIR433 gene (Chromosome 14: 100,881,886–100,881,978) [48] in iDCM patients showing the expansion of the differentially methylated CpG sites between iDCM and CNT. **b** Expression levels of the immature and mature form of hsa-miR-433-3p and its correlation with the LVEF. *p < 0.05; **p < 0.01. a.u., arbitrary units; CNT, control group; iDCM, idiopathic dilated cardiomyopathy; LVEF, left ventricular ejection fraction; TSS: transcription start site

To assess the potential clinical impact of epigenomic and transcriptomic alterations in iDCM patients, a correlation analysis was performed between an established echocardiographic parameter of cardiac function, LVEF, and differentially expressed and methylated miRNAs. This analysis revealed a significant simple correlation for hsa-miR-433-3p (*r* = 0.671, *p* value = 0.001) (Fig. 2).

## Discussion

The complex aetiology of iDCM and the critical need for effective patient management underscore the importance of advancing the understanding of its underlying pathological mechanisms [49]. In this context, miRNAs play a pivotal role in shaping the disease phenotype, contributing to its pathogenesis through a diverse range of mechanisms [50–54]. Therefore, unravelling the regulatory mechanisms behind their expression can be an important approach for elucidating their influence on iDCM. Among the various regulatory mechanisms of gene expression, DNA methylation has the ability to modify the accessibility of the transcriptional machinery, leading to changes in gene expression [55]. In this context, miRNAs may be influenced by specific methylation marks in the genome, which can alter their expression and, consequently, their ability to regulate cellular function [56]. Based on this idea, the present study aimed to investigate the relationship between the methylome and miRNA expression profiles by integrating transcriptomic and epigenomic analyses of LV samples from iDCM patients and CNT hearts. Our results revealed the existence of a subset of miRNAs with different expression patterns in both immature and mature forms that, additionally, presented different methylation states in the CpG positions of their predicted promoter regions. Moreover, one of these differentially expressed and methylated miRNAs, hsa-miR-433-3p was significantly correlated with LVEF, indicating a relationship between these modifications and the cardiac function.

The observed predominance of global hypomethylation among the identified DMPs derived from the methylome analysis aligns with our previous findings in ischaemic hearts [57], and with the reports of several other studies focused on other heart-related pathological conditions [58, 59]. In cancer, which is also characterized by a global hypomethylation of the tumoural tissue [60], the impact of these epigenetic patterns has been linked to various pathological mechanisms, including genetic instability and increased mutation rates [61], and some authors have linked an induction of inflammatory markers with hypomethylated genome [62]. Taken together, these findings may indicate that the observed global hypomethylation has a potential biological relevance in iDCM. However, mechanistic studies are required to determine whether this epigenetic pattern plays a causal role in disease pathogenesis.

When the results derived from the transcriptomic study were analysed, several of the identified differentially expressed mature miRNAs in iDCM patients were previously associated with other cardiac pathologies, such as arrhythmogenic cardiomyopathy [63, 64], congenital heart disease [65], acute heart failure [66, 67], and myocardial infarction [68]. In the context of DCM, previous studies have reported changes in the expression of hsa-miR-1-3p [69–71] and hsa-miR-499a-5p [72], which is consistent with our results. These findings support the hypothesis that specific miRNAs may play a functional role in modulating the cardiomyocyte phenotype during cardiac disease progression. Moreover, analysis of the corresponding pri-miRNAs revealed consistent differential expression in the precursors of 4 of the differentially expressed mature miRNAs. Notably, the expression patterns of all these pri-miRNAs were fully concordant with their respective mature forms, suggesting a coordinated transcriptional regulation. Interestingly, all of the differentially expressed pri-miRNA‒miRNA pairs presented upregulated expression in iDCM patients compared with controls.

The integration of both transcriptomic and epigenomic analyses revealed that a great majority of the differentially expressed pri-miRNAs contained at least one DMP within their defined promoter regions, which is consistent with previous reports highlighting the presence of CpG sites within miRNA promoters [44]. Further investigation into the relationship between methylation status and miRNA expression revealed that almost a 70% of the observed epigenetic modifications adhered to the conventional biological pattern [73], where hypermethylation is associated with transcriptional silencing and hypomethylation with overexpression. In contrast, some pri-miRNAs deviated from this expected trend, exhibiting hypermethylated DMPs linked to upregulated expression. The observed inconsistencies may be explained by two well-documented mechanisms: (i) miRNAs from methylated loci can be significantly downregulated following methylation removal and exhibit lower Drosha occupancy rates [74], and (ii) emerging evidence suggests that DNA methylation can, in certain contexts, contribute to transcriptional activation [75–78]. Nevertheless, other mechanisms may occur simultaneously, contributing to the observed alterations in pri-miRNA expression.

To further understand the functional relevance of these regulatory mechanisms in the context of iDCM, correlation analyses between the mature miRNA products and clinical variables were conducted, which revealed a significant positive correlation between LVEF and the levels of hsa-miR-433-3p. Given that a reduced LVEF is a hallmark clinical feature of iDCM [79], this correlation highlights a potential functional link between heart function, miRNA expression and epigenetic regulation. Additionally, these findings suggest that the upregulation of this miRNA in LV tissue from iDCM patients may represent a compensatory mechanism aimed at mitigating cardiac dysfunction. Interestingly, target prediction analyses for this miRNA support the potential regulatory role of DNA methylation in its expression by revealing interactions with genes relevant to the pathogenesis of iDCM. For hsa-miR-433-3p, a direct role in cardiac fibrosis has been previously described in three models of heart disease [80], indicating a potential involvement in determining iDCM pathogenesis.

The current study has several limitations that should be considered. First, the observational nature of the analysis and the small sample size limit its reliability, as it lacks experimental validation to reinforce the observed results. Despite this, our findings remain highly informative and valuable, given the unique nature of the LV tissue samples, and the challenges associated with their acquisition. Second, the use of a methylation array to assess epigenetic marks, while genome-wide, restricts the analysis to preselected CpG sites, which may exclude biologically significant regions, potentially affecting the results. Finally, the promoter regions identified using the miRStart 2.0 data may be subject to annotation inaccuracies, and findings could differ with more precise sequencing-based methods.

In summary, by integrating transcriptomic and epigenomic data, we identified a subset of differentially expressed miRNAs whose transcriptional activity appears to be influenced by DNA methylation patterns in their promoter regions. Moreover, one of these miRNAs, hsa-miR-433-3p, is significantly associated with cardiac dysfunction and myocardial fibrosis. These findings may contribute to a better understanding of the epigenetic mechanisms potentially involved in iDCM and suggest a possible role of DNA methylation in miRNA regulation. Further research is needed to validate these observations and explore their functional implications in disease progression, potentially paving the way for novel epigenetic-based therapeutic strategies.

## Supplementary Information

Additional file 1

Additional file 2

Additional file 3

Additional file 4

Additional file 5

## Data Availability

The data discussed in this publication have been deposited in NCBI's Gene Expression Omnibus [81] and are accessible through GEO Series accession number GSE307696 and GSE309058. All other supporting data from this study are available from the corresponding author upon reasonable request.

## Abbreviations

BP: Base pair
CNT: Control
CpG: Cytosine-guanine
DMP: Differentially methylated CpG position
DNMT: DNA methyltransferase
FC: Fold change
HF: Heart failure
iDCM: Idiopathic dilated cardiomyopathy
LVEF: Left ventricular ejection fraction
miRNA: MicroRNA
ncRNA: Noncoding RNA
pre-miRNA: Precursor miRNA
TSS: Transcription start site
